# Optimizing reconstruction parameters for quantitative ^124^I-PET in the presence of therapeutic doses of ^131^I

**DOI:** 10.1186/s40658-021-00398-z

**Published:** 2021-07-12

**Authors:** Louise M. Fanchon, Bradley J. Beattie, Keith Pentlow, Steven M. Larson, John L. Humm

**Affiliations:** 1 Department of Medical Physics, Memorial Sloan Kettering Cancer Center, 1275 York Avenue, New York, NY 10065 USA; 2 Department of Radiology, Memorial Sloan Kettering Cancer Center, 1275 York Avenue, New York, NY 10065 USA

**Keywords:** Quantitative ^124^I-PET, Targeted radionuclide therapy, Theragnostic

## Abstract

**Background:**

The goal of this work was to determine the quantitative accuracy and optimal reconstruction parameters for ^124^I-PET imaging in the presence of therapeutic levels of ^131^I. In this effort, images were acquired on a GE D710 PET/CT scanner using a NEMA IEC phantom with spheres containing ^124^I and increasing amounts of ^131^I activity in the background. At each activity level, two scans were acquired, one with the phantom centered in the field of view (FOV) and one 11.2 cm off-center. Reconstructions used an ordered subset expectation maximization algorithm with up to 100 iterations of 16 subsets, with and without time-of-flight (TOF) information. Results were evaluated visually and by comparing the ^124^I activity relative to the scan performed in the absence of ^131^I.

**Results:**

^131^I within the FOV added to the randoms rate, to dead time, and to pile-up within the detectors. Using our standard clinical reconstruction parameters, the image quality and quantitative accuracy suffered at ^131^I activities above 1.4 GBq. Convergence rates slowed progressively in the presence of increasing amounts of ^131^I for both TOF and nonTOF reconstructions. TOF reconstructions converged more quickly than nonTOF but often towards erroneous concentrations. Iterating nonTOF reconstructions to convergence produced quantitatively accurate images except for the off-center phantom at the very highest level of background ^131^I tested.

**Conclusions:**

This study shows that quantitative PET is feasible in the presence of large amounts of ^131^I. The high randoms fractions resulted in slow reconstruction convergence and negatively impacted TOF corrections and/or the accuracy of TOF information. Therefore, increased iterations and nonTOF reconstructions are recommended.

**Supplementary Information:**

The online version contains supplementary material available at 10.1186/s40658-021-00398-z.

## Background

Targeted radionuclide therapies (TRT) provide a unique opportunity for the concomitant use of theranostic radionuclide pairs to allow more precise patient-specific dosimetry measurements and to better assess treatment follow-up. For pre-therapy dosimetry, a diagnostic level of radiotracer is injected into the patient, followed by serial blood sampling and imaging [1, 2]. This information is used to estimate the patient dose distribution for the planned therapy with the assumption that the dose will scale linearly with the radioactivity injected. To validate this assumption in the context of radionuclide therapy with ^131^I-Iodide, we considered comparing pre-therapy PET images made using (the more quantitatively accurate) ^124^I-Iodide against similar PET images acquired following co-injection of ^124^I-Iodide along with the ^131^I-Iodide therapeutic dose. For this comparison to be valid, however, we first needed to determine if the presence of large amounts of ^131^I within and around the PET FOV impacts ^124^I quantitation.

Radioactive iodine (RAI) therapy with ^131^I is widely used to treat thyroid cancer [3]. ^131^I is also used clinically for other therapies such as recurrent or refractory neuroblastoma [4] with Iodine-131-m-iodobenzylguanidine (^131^I-mIBG), a structural analogue of a neurotransmitter. Successful and safe use of TRT requires knowledge of the radionuclide distribution in the patient: accurate quantification of the radiolabel uptake in the lesion and possibly other organs of interest. Since lesion uptake and biodistribution varies greatly from patient to patient, patients must be individually imaged. Knowing the patient-specific uptake of the radionuclide targeting agent allows for the customization of the therapeutic plan so that it is consistent with the dose constraints of the dose-limiting tissues [5].

Different approaches for RAI dosimetry have been used. Pacini et al. injected a low dose of ^131^I for pre-therapeutic SPECT scan imaging [6]. However, compared to PET, SPECT suffers from relatively low resolution and low sensitivity and has more severe count rate limitations. ^131^I emits high-energy gammas that penetrate the septa which further degrade image resolution, incur artifacts, and generally present challenges to quantitative imaging. Lower resolution is particularly problematic for small structures for which dose estimates are less accurate. Small lesions can be missed entirely. ^123^I has been used as an alternative to ^131^I, producing higher quality images afforded by the lower 160-keV photon emission energy and at lower radiation dose per unit administered activity. However, the 13.2-h half-life of ^123^I limits the ability to perform late time point imaging, important for the estimation of the dosimetry for many radiopharmaceuticals.

To rectify these deficiencies, the positron-emitter ^124^I has been proposed for pre-therapy lesion dosimetry [7]. ^124^I has a 4.2-day half-life and allows for quantitatively accurate PET imaging, making it well suited for radioiodine dosimetry applications. Beijst et al. studied the difference in lesion detectability between ^131^I-SPECT and ^124^I-PET imaging and found that ^124^I-PET offers better image quality for similar activity concentrations [8, 9].

The clinical value of pre-therapeutic dosimetry is still being debated due to the uncertainty surrounding the assumption of linearity of uptake between the pre-therapeutic and therapeutic doses. It is possible that the radioiodine tracer dose given before therapy might change the tissue’s avidity for radioiodine or the radiolabeled carrier molecule. This effect could be pharmacologic or potentially radiologic with the latter often referred to as “stunning”. Quantitative ^124^I-PET before and during therapy could provide new information on the accuracy of pre-therapy dosimetry.

A few phantom studies investigating the ability to image ^124^I in the presence of ^131^I have been published. Lubberink et al. looked at optimizing acquisition parameters (energy window) for 2-D and 3-D ^124^I-PET imaging with 75 MBq of ^131^I in the background of an IEC phantom, based on images acquired on an ECAT EXACT HR + scanner (CTI/Siemens) [10]. They observed a loss in image quality compared to ^124^I-PET alone but did not report on ^124^I quantitative accuracy and these results cannot be extrapolated to newer scanners. Braad et al. [11] measured the accuracy of ^124^I-PET on a GE D690 PET/CT scanner, a scanner in the same class as the D710 PET scanner used here. Their study, however, focused on contrast recovery using a variety of sphere to background ratios in data sets reconstructed using a single set of reconstruction parameters, while in our study we focused on the impact that the presence of ^131^I had on the reconstruction convergence rate. Also, the maximum amount of ^131^I they considered was 1.25 GBq whereas we sought to determine the full range of background ^131^I levels over which quantitative accuracy would be maintained and used up to 3.7 GBq of ^131^I.

## Methods

### Iodine isotopes

^131^I decays by beta minus emission, with an 8.02-day half-life, making it well suited for radionuclide therapy applications. In 81% of decays, a 364-keV gamma is also emitted and in ~ 7% a 637-keV gamma is produced. Although there are no coincident emissions among these gammas, they fall within the energy window (425–650 keV) used by the PET camera to detect the 511-keV annihilation photons. Given large enough quantities of ^131^I within or near the PET FOV, these will add considerably to detector dead time and pile-up and can produce a significant number of random coincidence events, adding both noise and potential quantitative bias to the PET images.

^124^I decays 77% by electron capture and 23% by positron emission. The positron emission allows imaging of ^124^I distribution with PET; however, half of the positrons are emitted in a cascade that includes a 602.7-keV prompt gamma (i.e., a gamma produced within the timing window used to identify annihilation photon coincidence pairs). The energy of this gamma is within the PET energy window, so coincidences between the prompt gamma and an annihilation photon cannot be differentiated from those resulting from an annihilation photon pair, resulting in spurious “cascade coincidences” that contribute to an additional background signal which affects the quantification if left uncorrected [12, 13].

### PET/CT camera

All scans were acquired on a lutetium-yttrium orthosilicate (LYSO) GE Discovery 710 PET/CT scanner (GE Medical Solutions, Waukesha, WI, USA). This camera has a 15.7 cm axial by 70.0 cm FOV, timing resolution of 560 ps, and a sensitivity of 7.5 cps/kBq when using an energy window of 425–650 keV and a 4.9-ns timing window. Dead time is determined based on a directly measured block busy signal. Scatter correction is based on a distribution calculated using a single scatter model, the amplitude of which is determined via tail-fit. Cascade coincidences are accounted for by including an additional constant parameter when performing the tail-fit of the modeled scatter correction. The use of a constant during this fit assumes that the cascade coincidence distribution is uniform throughout the projection space. All reconstructions were performed off-line using Release 2.0 of GE’s PET Reconstruction Toolbox made available to us through a research study agreement.

### Phantom

PET scans were performed using a NEMA IEC body phantom (24.1 × 30.5 × 24.1 cm, 9.7 L) containing a lung insert and six fillable spheres of various diameters: 10, 13, 17, 22, 28, and 37 mm. All spheres were filled with 0.37 MBq/cc of ^124^I (a concentration typically seen in thyroid cancer lesions given the planned pre- and mid-therapy ^124^I injected doses). A baseline PET scan was acquired with a cold (i.e., no radioactivity) water-filled background. Subsequent PET images were acquired with increasing amounts of ^131^I in 740 MBq increments up to 3.7 GBq. For each scan, the phantom was placed back in the same position using the laser alignment lines and markers on the PET table. PET scans at each background activity level were acquired with the table at a height that centered the lung insert within the FOV and again with the table raised to its maximum height (11.2 cm higher), resulting in “off-center” images. These same two table heights were used for all background activity levels, resulting in two groups of scans (centered and off-center). The off-center phantom was included in this study because we anticipated that it would incur non-uniform randoms and cascade coincidence distributions and that this might prove especially challenging both to the PET camera hardware and image reconstruction process.

### PET/CT acquisition and reconstruction

Scans were acquired in time-of-flight mode, for 8 min using a single bed position centered over the axial extent of the phantom. PET images were reconstructed using our standard clinical reconstruction protocol: a TOF 3D-ordered subset expectation maximization (OSEM) reconstruction algorithm with 2 iterations, 16 subsets, point-spread function modeling (GE SharpIR), a 128 × 128 × 47 matrix (voxel spacing of 5.47 × 5.47 × 3.27 mm), 1-2-1 axial smoothing, 6.4 mm Gaussian in-plane smoothing, and random coincidences estimated from singles. Additional image reconstructions using up to 100 iterations were performed with and without TOF information. The CT scan parameters were 120 kV, 70 mA, 700 mm FOV, 1.25 mm slice thickness, and 512 × 512 matrix size.

### Data analysis in image space

Centered and off-center scans were registered to one another using rigid body transforms (HybridViewer v4.0.0, Hermes Medical Systems). Spherical volumes of interest (VOIs) of 5.5 cm in diameter, were drawn on the baseline scan centered over each sphere containing ^124^I. A seventh spherical VOI was placed in the background. All VOIs were copied and applied to the other image sets. VOIs around the spheres were drawn with large margins to capture all the radioactivity emanating from each sphere (see Supplemental Figure S1). Because there was no ^124^I in the background region, each sphere’s activity concentration can be determined by taking the total activity inside the VOI and dividing it by the sphere’s physical volume. For spheres containing air bubbles, the volume was adjusted after measuring the air bubble volume on the CT image. ^124^I activity concentration was decay-corrected to the time of the first scan’s acquisition.

To assess convergence rates, concentration measurements were made using a small VOI (3 × 3 × 3 voxel) placed in the approximate center of each sphere and applied to images reconstructed with between 1 and 100 iterations. Each of these curves was then normalized by its final value.

### Data analysis in projection space

Region of interest (ROI) measurements were taken from the line-of-response (LOR) data in nonTOF projection space (i.e., projections in which the TOF bins have been summed together). In these measurements, only the LORs perpendicular to the scanner axis were included (i.e., the oblique angles were excluded). Each ROI included the 25 LORs (block of 5 × 5 pixels) passing through the approximate center of the largest sphere. The position of the ROI was adjusted so that it traced a sinusoidal path as a function of the LOR ray angle (see video in Supplemental data S2) and the mean over all angles was measured. In addition, an ROI of identical size and placement as a function of angle but offset in the axial dimension was used to sample only background activity (i.e., excluding activity from hot spheres). Measurements were taken from the raw prompts, random coincidences (estimated from singles), scattered coincidences (modeled), raw true coincidences (prompts minus random and scatter), and fully corrected true coincidence projection data sets (i.e., trues corrected for dead time, pile-up, and attenuation).

## Results

### Activity quantification when using clinical reconstruction parameters

**Fig. 1:**
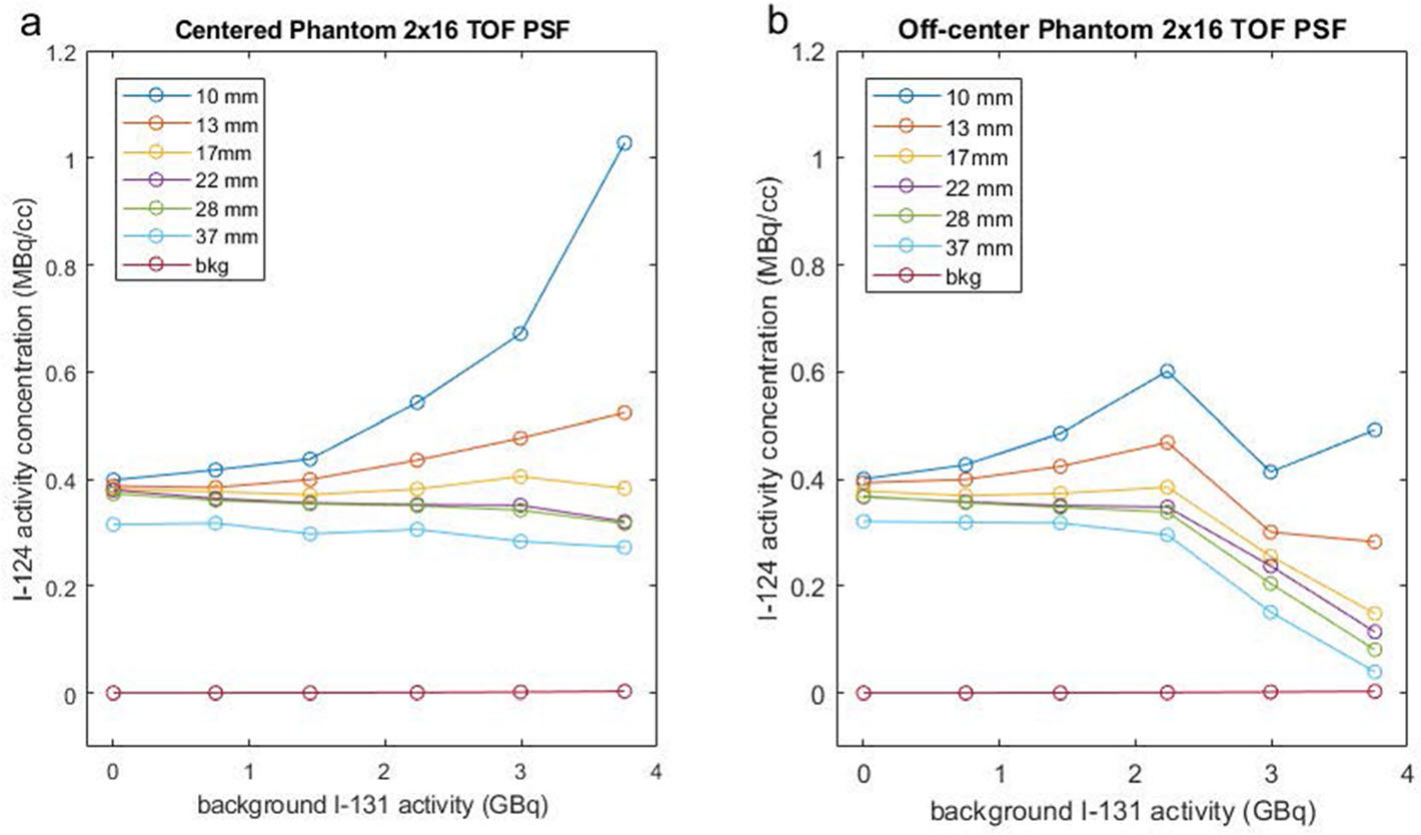
^124^I activity concentration in the phantom’s spheres as a function of increasing ^131^I activity in the background measured on the PET image reconstructed with the OSEM algorithm with 2 iterations and 16 subsets, TOF information and PSF modeling. The phantom was placed in the middle of the field of view (**a**) and on the scanner table at the anterior most position, 11.2 cm higher (off-center) (**b**)

The initial measurements of the ^124^I activity concentration were based on PET images reconstructed using our standard clinical reconstruction parameters, 2 iterations of the OSEM algorithm utilizing 16 subsets (2 × 16) in TOF-mode with point-spread modeling (SharpIR). Based on these images, the apparent activity in the spheres as a function of ^131^I background activity differed depending both on sphere size and the phantom’s position within the FOV (Fig. 1a, b). For the centered scans and for increasing background activity, apparent activity concentration tended to decrease slightly in the larger spheres, whereas it increased dramatically in the smaller spheres. The activity increase for the small spheres was attributable to the oversized VOIs we drew to capture all the activity emanating from each sphere assuming that the true background was zero. The results here suggest that the apparent background was above zero in these images. For the largest sphere, the seeming low activity is likely due to activity not captured within the VOI because of partial voluming (i.e., the VOI was not oversized enough, having only 0.4-cm excess border for the 3.7-cm-diameter sphere). For the off-center scans, the measured activity in the four largest spheres was stable up to 2.2 GBq of ^131^I, but dropped precipitously for higher activities. For the two smallest spheres, the measured activity was quite variable, at times either increasing or decreasing in the presence of additional background ^131^I. The small spheres were difficult to visualize at ^131^I activities above 2.2 GBq.

### Relative activity quantification taken in projection space

**Fig. 2:**
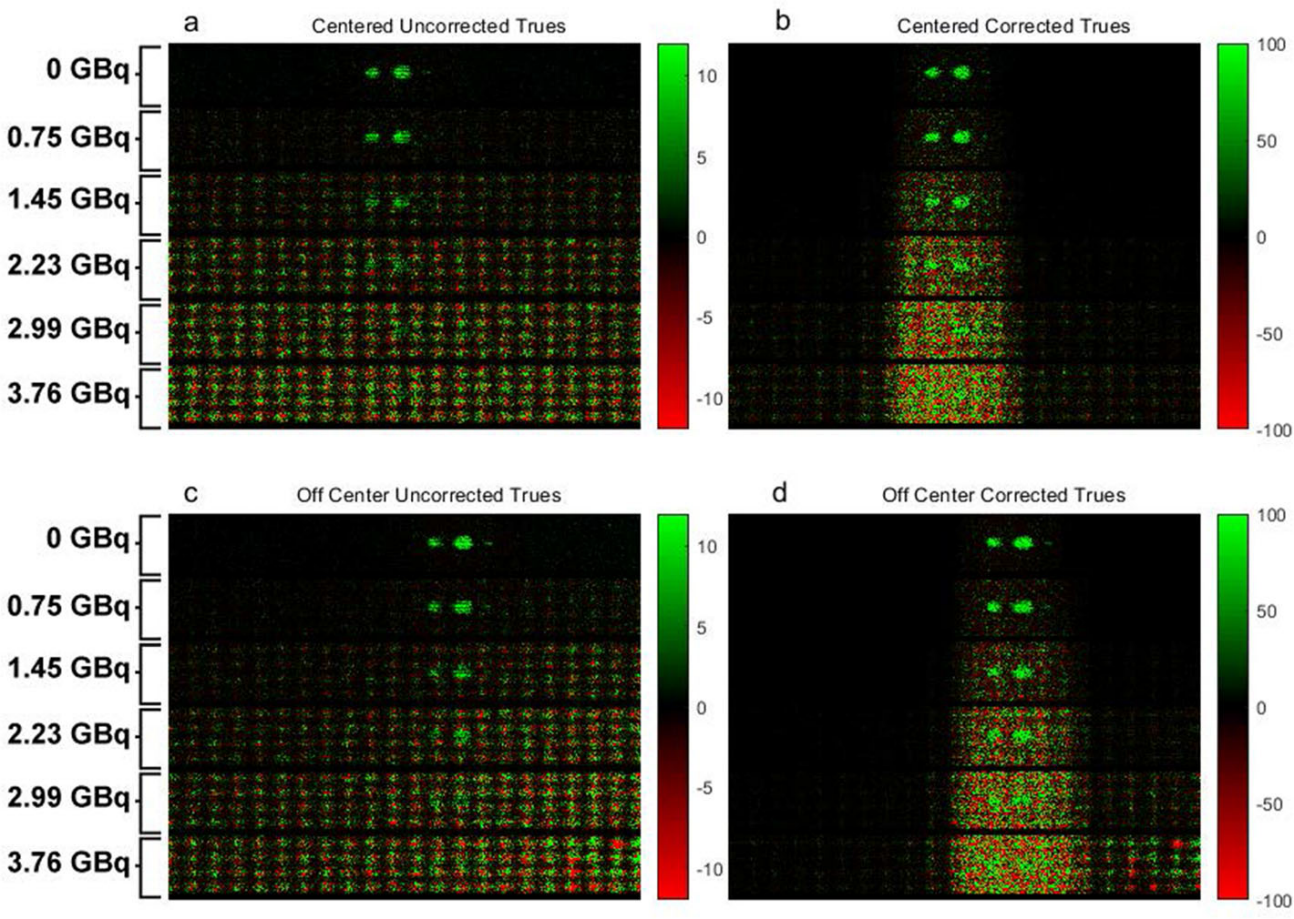
Projection images of the number of uncorrected true events (**a**, **c**) and corrected true events (**b**, **d**) for the centered (**a**, **b**) and off-centered phantom (**c**, **d**) acquisition and for increasing ^131^I activity in the background. The corrected number of trues corresponds to the number of trues recorded by the PET system multiplied by the dead-time correction factor, the normalization factor, and the attenuation correction factor

The source of the quantitative errors seen at high ^131^I background levels was determined by taking measurements from the raw projection data with and without various corrections and from the random and scatter coincidence corrections themselves. Examples of this data are shown in Fig. 2 which shows a single projection angle of the raw LOR “true” coincidences (Fig. 2a, b) and with all corrections applied (Fig. 2c, d). Although the spheres sometimes overlapped depending on the angle of the projection (and thus the measurements cannot be said to be always of a single sphere), equivalently made measurements still allow for comparisons among the scans with a differing background.

**Fig. 3:**
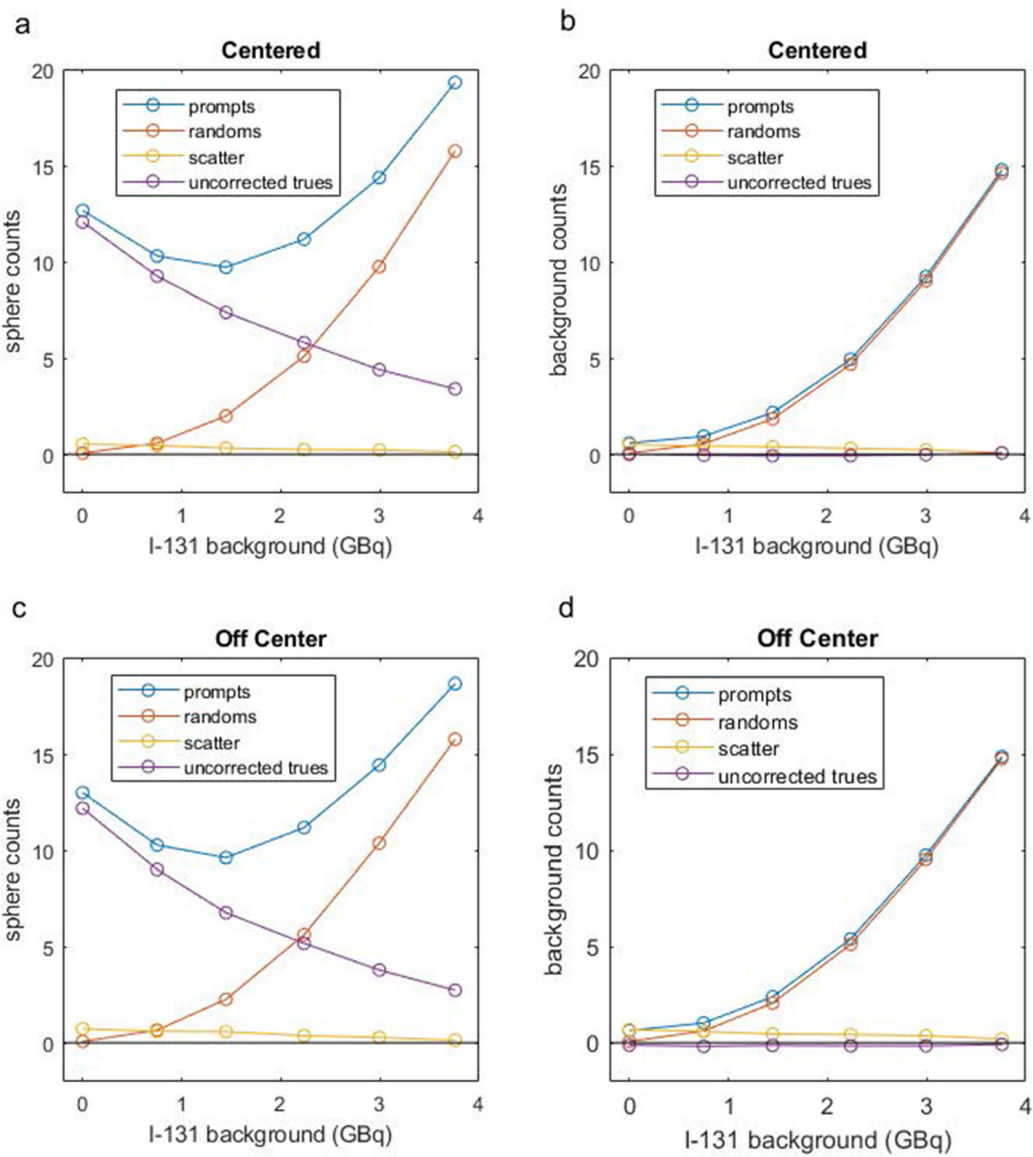
Number of uncorrected true, scatter, and random events measured in a small VOI placed at the center of the 37-mm-diameter phantom sphere (**a**, **c**) and in the background (**b**, **d**) in projection space and for all angles as a function of increasing ^131^I activity in the background for acquisitions with the phantom centered (**a**, **b**) and off-centered (**c**, **d**)

As can be appreciated from Fig. 3a and c (centered and off-center scans, respectively), the random coincidences measured from the LORs passing through the largest sphere increase approximately with the square of the ^131^I activity, overtaking the uncorrected true coincidences at 2.2 GBq and ultimately outnumbering them by several fold. Above 2.2 GBq of ^131^I, the true and scattered coincident events decrease consistently with the increasing dead time. Counts sampled from the LORs passing through the background region (Fig. 3b, d) show random and scattered coincidence levels similar to that in Fig. 3a and c. The true coincidences, however, are appropriately near zero regardless of the background ^131^I activity level suggesting that the randoms and scatter are accurately estimated in the nonTOF projection space.

**Fig. 4:**
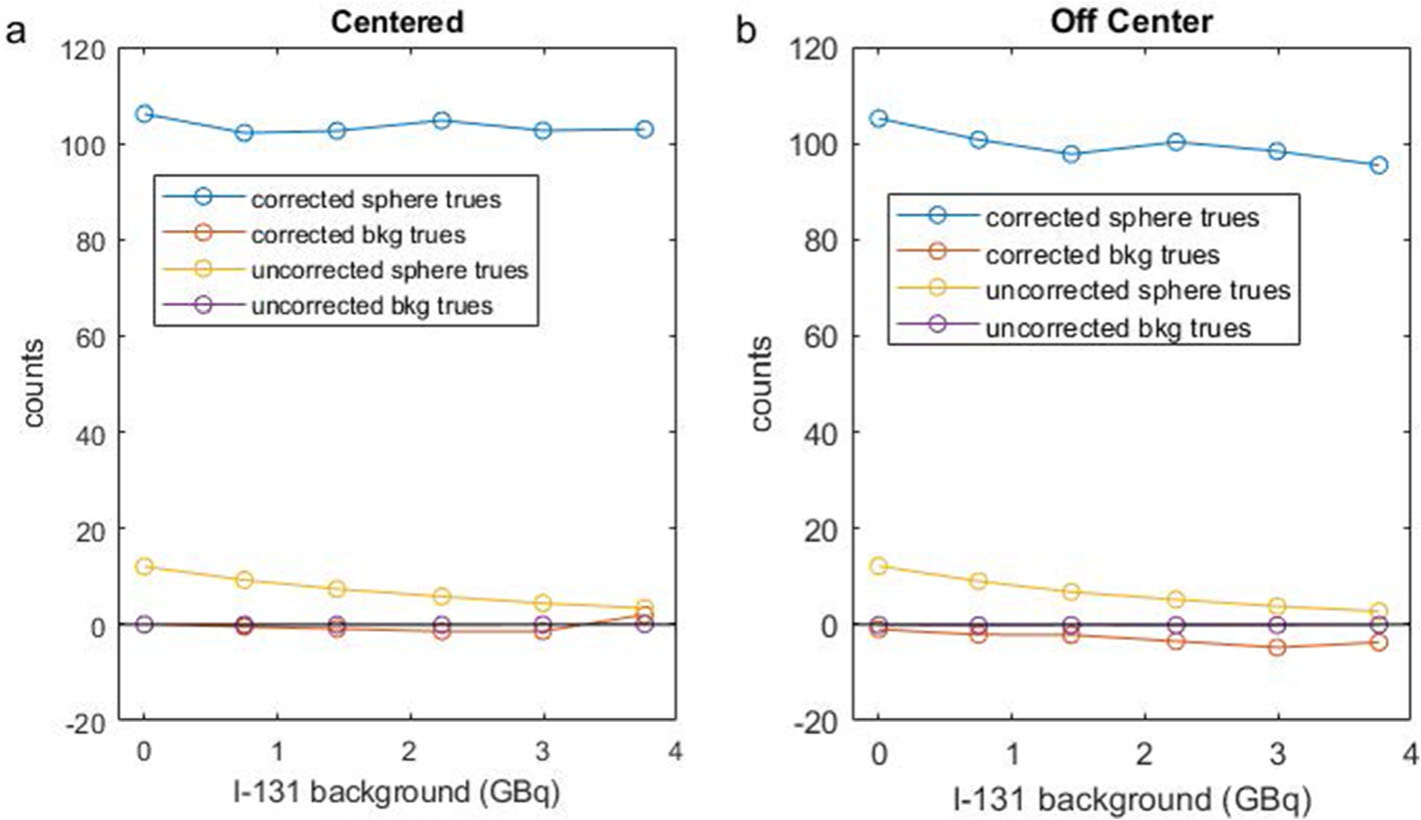
Corrected and uncorrected number of true events measured in projection space at the center of the 37-mm-diameter sphere of the IEC phantom and in the phantom background, for increasing ^131^I activity concentration and for acquisition performed with the phantom centered in the FOV (**a**) and the phantom off-centered (**b**)

Figure 4a (centered phantom) shows that the dead-time correction is functioning properly, yielding an essentially constant level of true counts from the LORs passing through the large sphere for all ^131^I activity levels (note: the attenuation correction has been applied but is not impacted by ^131^I activity). In the off-center phantom, the corrected trues have a slight decline with increasing ^131^I activity (Fig. 4b).

### Rate of convergence

**Fig. 5:**
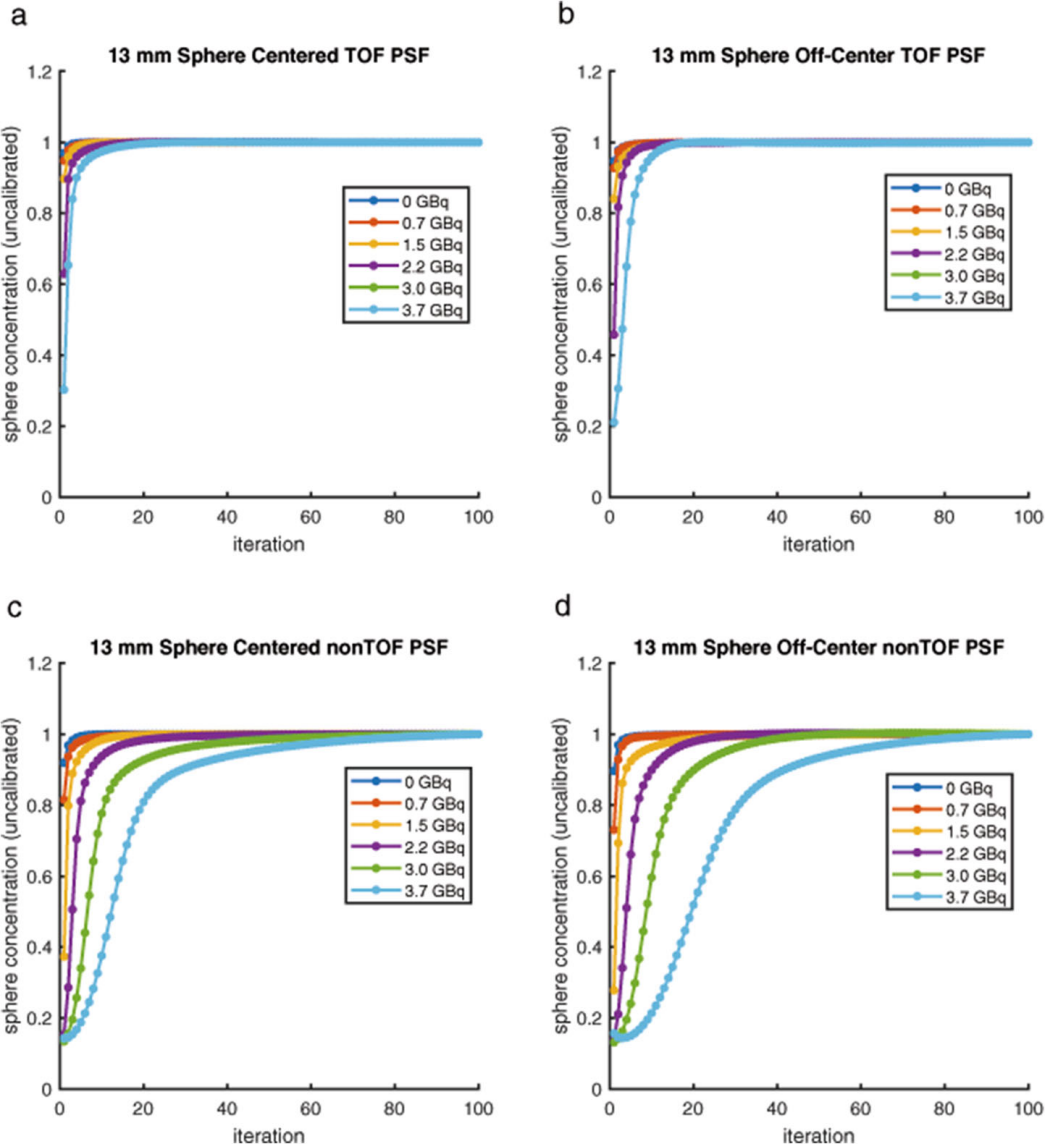
Normalized mean ^124^I concentration measured on the PET image, in a small (3 × 3 × 3 voxel) VOI at the approximate center of the 13-mm-diameter sphere for the centered phantom reconstructed with TOF information (**a**), for the off-center phantom reconstructed with TOF information (**b**), for the centered phantom reconstructed without TOF information (**c**), and for the off-center phantom reconstructed without TOF information (**d**) all shown as a function of the number of iterations. This measurement was done for scans acquired with 0, 0.75, 1.45, 2.23, 2.99, and 3.76 GBq of ^131^I in the background. Each curve has been normalized to the concentration seen after 100 iterations

After applying all corrections, there was a constant number of counts emanating from the ^124^I-filled spheres in projection space across all background levels. We therefore were left to explain why the post-image reconstruction results were not also constant (Fig. 1a, b) and so chose to investigate the convergence rate for the various spheres as impacted by the ^131^I background activity level. The plots in Fig. 5a–d show that increasing amounts of ^131^I background activity cause a marked reduction in the rate of convergence of the iterative image reconstruction algorithm overall. Reconstructions using TOF information converged more quickly than nonTOF reconstructions and for the off-center phantom convergence rates were slower, especially for the smallest and furthest off-center spheres (Supplemental Figures S3 and S4). For both the centered and off-center phantoms with background ^131^I activity levels up to 0.7 GBq, two iterations were enough to achieve concentrations close to the final value. With larger amounts of ^131^I in the background (and especially for the off-center phantom), stopping at two iterations incurred large errors. Overall, 25 iterations of the TOF reconstruction algorithm were necessary to reach convergence in all regions of the centered phantom and 75 iterations were required for nonTOF reconstruction.

### Activity quantification when using high number of iterations

**Fig. 6:**
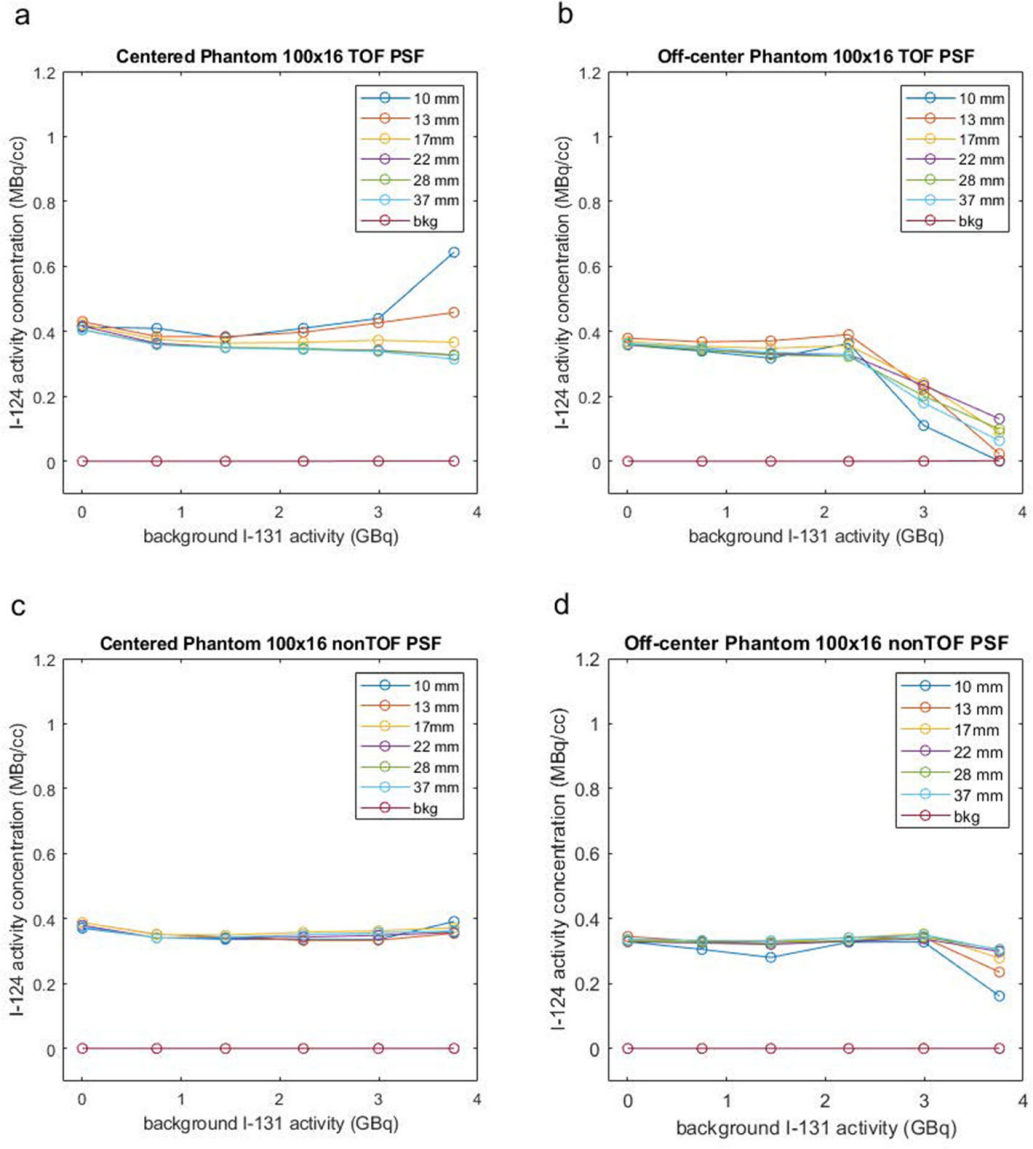
^124^I activity concentration in the phantom’s spheres as a function of increasing ^131^I activity in the background measured on the PET image reconstructed with the OSEM algorithm with 100 iterations and 16 subsets. In **a**, the phantom was centered and reconstructed with TOF information. In **b**, the phantom was off-center and reconstructed with TOF information. In **c**, the phantom was centered and reconstructed without TOF information. And in **d**, the phantom was off-center and reconstructed without TOF information

To circumvent these convergence-related inaccuracies, we analyzed the quantitative accuracy of images reconstructed with and without TOF information at 100 iterations of 16 subsets. ^124^I-PET images reconstructed with TOF and 100 iterations were more quantitatively accurate than images reconstructed with 2 iterations (Fig. 6a, b) except for the off-centered scans with^131^I background activity above 3.0 GBq.

The results for the nonTOF reconstructions using 100 iterations show that the nonTOF reconstructions outperformed the TOF reconstructions in quantitative accuracy (Fig. 6c, d). For the centered phantom, the nonTOF reconstruction remained accurate regardless of the level of ^131^I background. For the off-center phantom, however, there was a slight decrease in apparent activity seen in all spheres at 3.7 GBq of ^131^I activity.

**Fig. 7:**
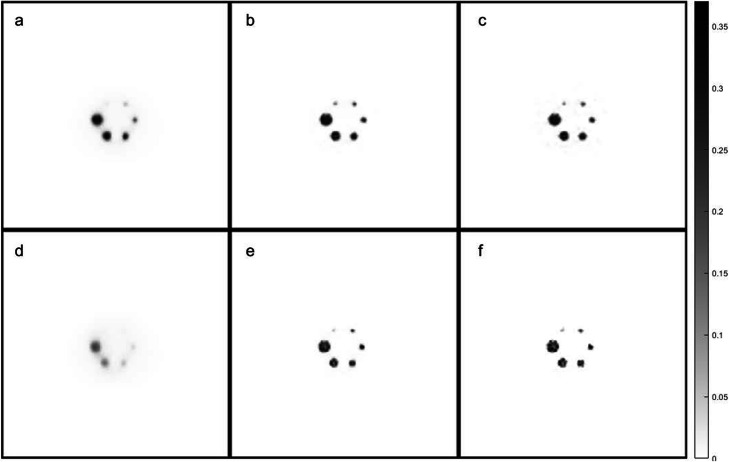
Transverse slice of the IEC phantom through the center of the hot spheres acquired with the phantom in the center position with 3.76 GBq of ^131^I in the background. The images on the top row (**a**–**c**) were reconstructed with OSEM using TOF information with 16 subsets and 2, 25, and 100 iterations, respectively. In the second row (**d**–**f**), images were similarly reconstructed but without making use of TOF information

Figure 7 shows the impact of iteration number on image quality for both the TOF (top row) and nonTOF (bottom row) reconstructions of the centered phantom having 3.7 GBq of ^131^I in the background. As the number of iterations is increased, conspicuity of the spheres (especially for the smallest) improves considerably although the noise increases slightly and the background level is reduced. Some spurious background activity remains especially for the TOF case (Supplemental Figure S5). For the off-center phantom, however, image quality for the TOF reconstructions was clearly degraded at the higher ^131^I activity levels compared to the nonTOF reconstructions (Supplemental Figure S6).

## Discussion

The results of this study show that quantitative ^124^I-PET imaging can be done in the presence of therapeutic amounts of ^131^I on a GE D710 scanner if more iterations are used during the reconstruction. Our typical clinical image reconstructions are performed with TOF data using an OSEM algorithm involving 2 iterations and 16 subsets and PSF modeling. We found that with increasing levels of ^131^I in the phantom’s background, incomplete convergence resulted in quantitative errors and low image contrast. Increasing the number of iterations improved image quality and quantitative accuracy for both TOF and nonTOF reconstructions, but the number of iterations necessary was beyond what is typically used clinically and increased with increasing ^131^I levels.

We speculate that the overall slowing of the convergence rate is due to the extraordinarily high per-crystal singles rates caused by the ^131^I within these data sets (up to 42 kcps—see supplemental figure S7). High per-crystal singles rates, in turn, result in high numbers of random coincidences. It is typical (and true of the GE reconstruction algorithm) to add random coincidences and other corrections to the estimated true coincidences within each iterated loop of the reconstruction prior to taking the ratio relative to the measured prompt coincidence data in order to preserve the Poisson noise model assumed in OSEM reconstruction. It is this ratio that is sampled and used to update the image estimate during the backprojection step of the OSEM algorithm. When the randoms far exceed the trues, as they do here, this is effectively equivalent to adding a large number to two small numbers, before taking their ratio. Because the updated ratios tend to be much closer to unity, they slow convergence. We tested this hypothesis by artificially adding a constant (of magnitude similar to the 3.7 GBq ^131^I per LOR randoms) to both the measured prompts and estimated randoms projection data for the centered phantom with no ^131^I in the background (data not shown) and this caused a similar delay in convergence.

Most surprising, however, was that the nonTOF reconstructions outperformed the TOF reconstructions at the highest levels of ^131^I background. Generally, the use of TOF information during reconstruction would be expected to speed convergence and reduce noise in the images, essentially without a downside. But here we find that either the TOF information is itself compromised or the TOF-specific corrections for the various PET confounds are somehow becoming problematic under these conditions.

The ^131^I gammas, in addition to greatly increasing randoms, also increase the dead time (the mean dead-time correction factor per pixel increased from 1.02 without background activity to 3.78 for the off-center scan with 3.7 GBq of ^131^I in the background) and cause a high degree of pile-up in all detector blocks. This pile-up, in turn, could conceivably negatively impact the TOF measurement, though we did not test this directly. The pile-up can best be appreciated by looking at the raw prompt counts in projection space (supplemental Figure S8a and b). Pile-up causes the scintillation events occurring within a PET detector block to tend to appear to be positioned at the center of the block. Thus, each of the “dots” discernable in supplemental figure S8 corresponds to a detector block. It is also worth noting that both pile-up and the overall random events, prompts, and dead time are not uniform when the phantom is positioned off-center (see comparison between supplemental Figure S8a and b).

The GE D710 image reconstruction includes a correction for pile-up which along with the dead time and other corrections appears to have worked remarkably well in this data. However, small inaccuracies in these large corrections might explain the inaccuracies seen particularly in the off-center phantom at the highest ^131^I background level. For example, Fig. 4b shows that the average corrected number of trues over a region of the background is slightly negative. The number of trues being defined as the number of prompts minus random and scatter, if the number of estimated trues is negative (which does not have a physical reality), it suggests an overcorrection of the number of scatter and/or random events in that region. We hasten to point out that this was only true of the off-center phantom and it would be very unlikely to encounter a clinical data set this poorly placed. Similarly, we have estimated that each level of ^131^I that we placed within the phantom background corresponds to roughly 4 times that amount within a patient, thus the highest level of ^131^I we tried (3.7 GBq) is unlikely to be encountered clinically.

By showing that quantitative ^124^I-PET is feasible in the presence of large amount of ^131^I in the PET FOV, we laid the groundwork for investigating the uptake linearity between pre-therapeutic and therapeutic doses in RAI. Radioiodine therapies can sometimes involve the administration of up to 15 GBq (400 mCi) of ^131^I. We do not know whether the uptake of radioactive iodine predicted from a pre-therapeutic tracer dosimetry study accurately predicts the uptake for these high-dose therapies. This study demonstrates the feasibility of using ^124^I-PET to perform image-derived lesion dosimetry during the actual therapy and confirm the predictions of pre-therapeutic radiotracer studies. In addition, such a capability would allow us to conduct a rigorous study of radioiodine thyroid lesion stunning (i.e., significant reduction in radioiodine uptake following the administration of a tracer), in lesions where this phenomenon is observed.

## Conclusions

The results of this study show that concomitant ^124^I-PET imaging for patients undergoing ^131^I therapy is feasible and should provide accurate activity quantification and satisfactory image quality for activities up to 3.76 GBq of ^131^I in the background. This result likely extends to other PET models from GE that have the same FOV size and share its method of dead-time determination and correction. The corrections applied by the data prior to reconstruction appear to be accurate but noise in the data appears to slow the rate of convergence during image reconstruction and using TOF information tended to reduce quantitative accuracy compared to nonTOF reconstructions, when both were fully converged. Increasing the number of iterations above what is typically used clinically greatly improves quantitative accuracy and image quality.

## Supplementary Information

**Additional file 1: Figure S1.** Image show VOI placement. Each circle represents a 5.5 cm diameter spherical VOI placed to include virtually all the activity emanating from the enclosed physical sphere despite partial volume affects. Activity concentrations within the spheres were calculated by dividing the total activity measured within each VOI by the volume of the physical sphere corrected for any small air bubbles. For the background VOI, the mean value was taken.

**Additional file 2: Figure S2.** Video showing placement of ROIs used to sample large sphere in projection space for centered phantoms.

**Additional file 3: Figure S3.** Normalized mean ^124^I concentration measured on the PET image, in a small (3x3x3 voxel) VOI at the approximate center of the 10 mm (A), 13 mm (B), 22 mm (C), 28 mm (D), 37 mm (E) diameter sphere for the phantom imaged off-center and reconstructed without TOF information shown as a function of the number of iterations. This measurement was done for scans acquired with 0, 0.75, 1.45, 2.23, 2.99, 3.76 GBq of ^131^I in the background. Each curve has been normalized to the concentration seen after 100 iterations.

**Additional file 4: Figure S4.** Same as Fig. S3 except for a centered phantom.

**Additional file 5: Figure S5.** Background activity measured on the ^124^I PET image of the NEMA IEC phantom reconstructed with the OSEM algorithm with 2 and 100 iterations, as a function of increasing activity of ^131^I in the background and for the phantom centered and off centered within the field of view, using TOF information in (**A**) and without TOF information in (**B**).

**Additional file 6: Figure S6.** Transverse slice of the IEC phantom through the center of the hot spheres acquired with the phantom in the off-center position. The images on the top row (**A**, **B**, **C**) were reconstructed with OSEM using TOF information with 16 subsets and 100 iterations while the data was acquired with 2.2, 3.0 and 3.7 GBq of ^131^I in the background, respectively. In the second row (**D**, **E**, **F**) images were similarly reconstructed but without making use of TOF information.

**Additional file 7: Figure S7.** The maximum singles count rate of any crystal as a function of ^131^I in the phantom when centered in the FOV and when 11.2 cm off-center.

**Additional file 8: Figure S8.** Projection image of the number of prompts for increasing ^131^I activity, for centered (**A**) and off-centered (**B**) acquisitions.

## Data Availability

This study involved measurements with phantom only and contains no clinical data. All the image data and analysis results are presented in the manuscript. The original PET/CT images will be made available upon request.

## Abbreviations

FOV: Field of view
GE: General Electric
131I-mIBG: Iodine-131-m-iodobenzylguanidine
LOR: Line of response
LYSO: Lutetium-yttrium oxyorthosilicate
NEMA IEC: National Electrical Manufacturers Association and International Electrotechnical Commission
OSEM: Ordered subset expectation maximization
PET: Positron emission tomography
RAI: Radioactive iodine
ROI: Region of interest
TOF: Time of flight
TRT: Targeted radionuclide therapies
VOI: Volume of interest
