# Supplementary figures and images for: Optimizing reconstruction parameters for quantitative 124I-PET in the presence of therapeutic doses of 131I
Source: EJNMMI Phys. 2021 Jul 12;8:50. doi: 10.1186/s40658-021-00398-z (PMC8273044; doi:10.1186/s40658-021-00398-z)

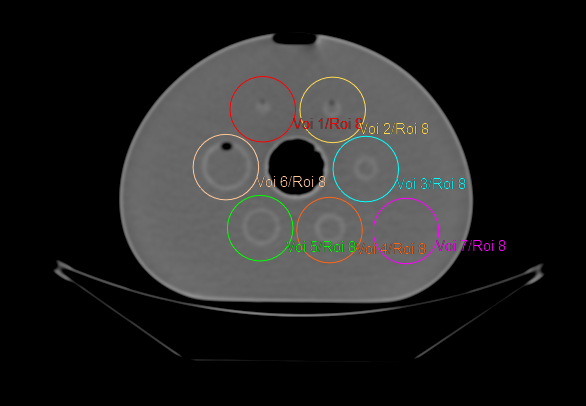

Supplement: Supplementary file 1 — Additional file 1: Figure S1. Image show VOI placement. Each circle represents a 5.5 cm diameter spherical VOI placed to include virtually all the activity emanating from the enclosed physical sphere despite partial volume affects. Activity concentrations within the spheres were calculated by dividing the total activity measured within each VOI by the volume of the physical sphere corrected for any small air bubbles. For the background VOI, the mean value was taken. [file 40658_2021_398_MOESM1_ESM.png]

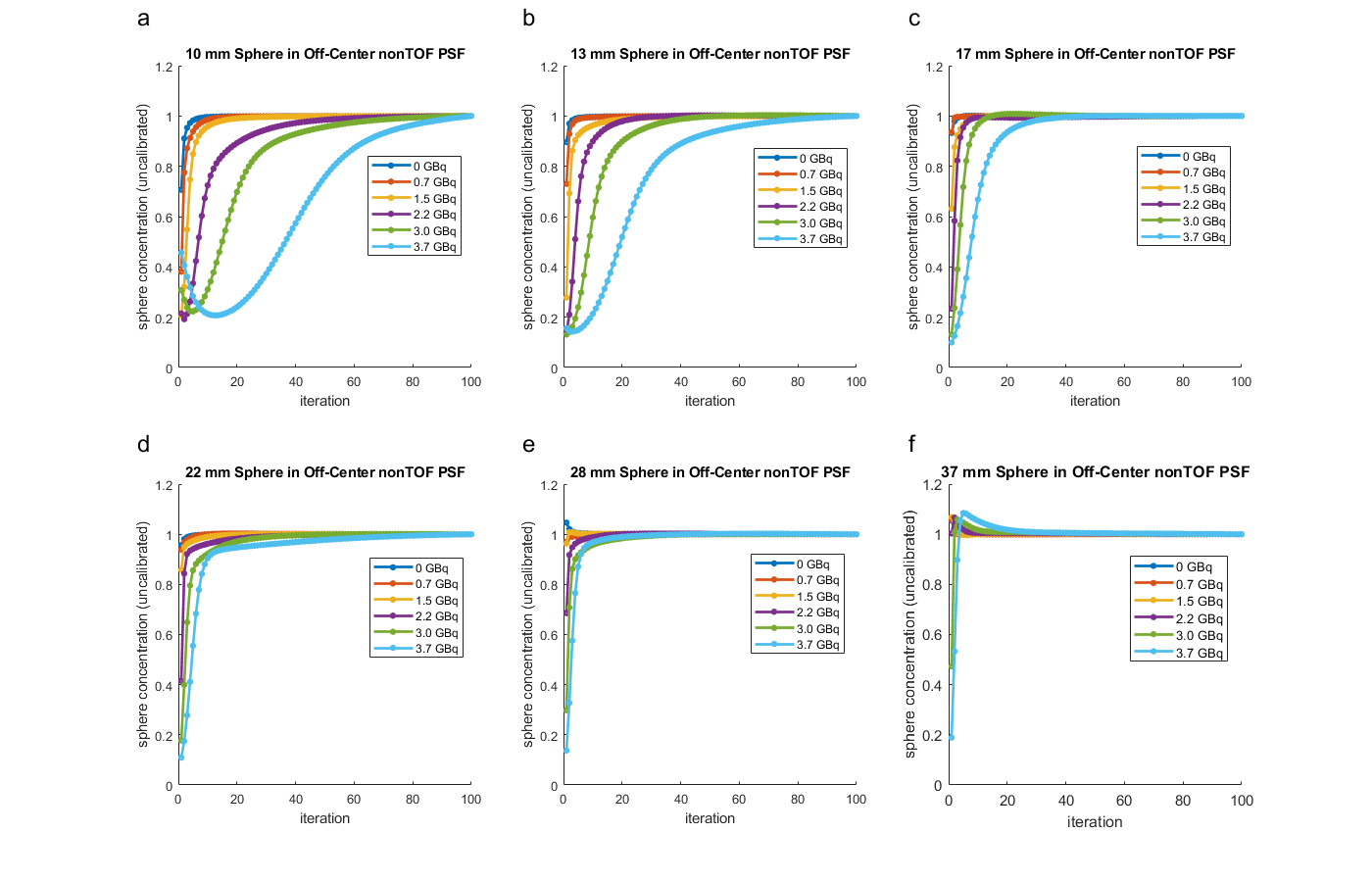

Supplement: Supplementary file 3 — Additional file 3: Figure S3. Normalized mean 124I concentration measured on the PET image, in a small (3x3x3 voxel) VOI at the approximate center of the 10 mm (A), 13 mm (B), 22 mm (C), 28 mm (D), 37 mm (E) diameter sphere for the phantom imaged off-center and reconstructed without TOF information shown as a function of the number of iterations. This measurement was done for scans acquired with 0, 0.75, 1.45, 2.23, 2.99, 3.76 GBq of 131I in the background. Each curve has been normalized to the concentration seen after 100 iterations. [file 40658_2021_398_MOESM3_ESM.png]

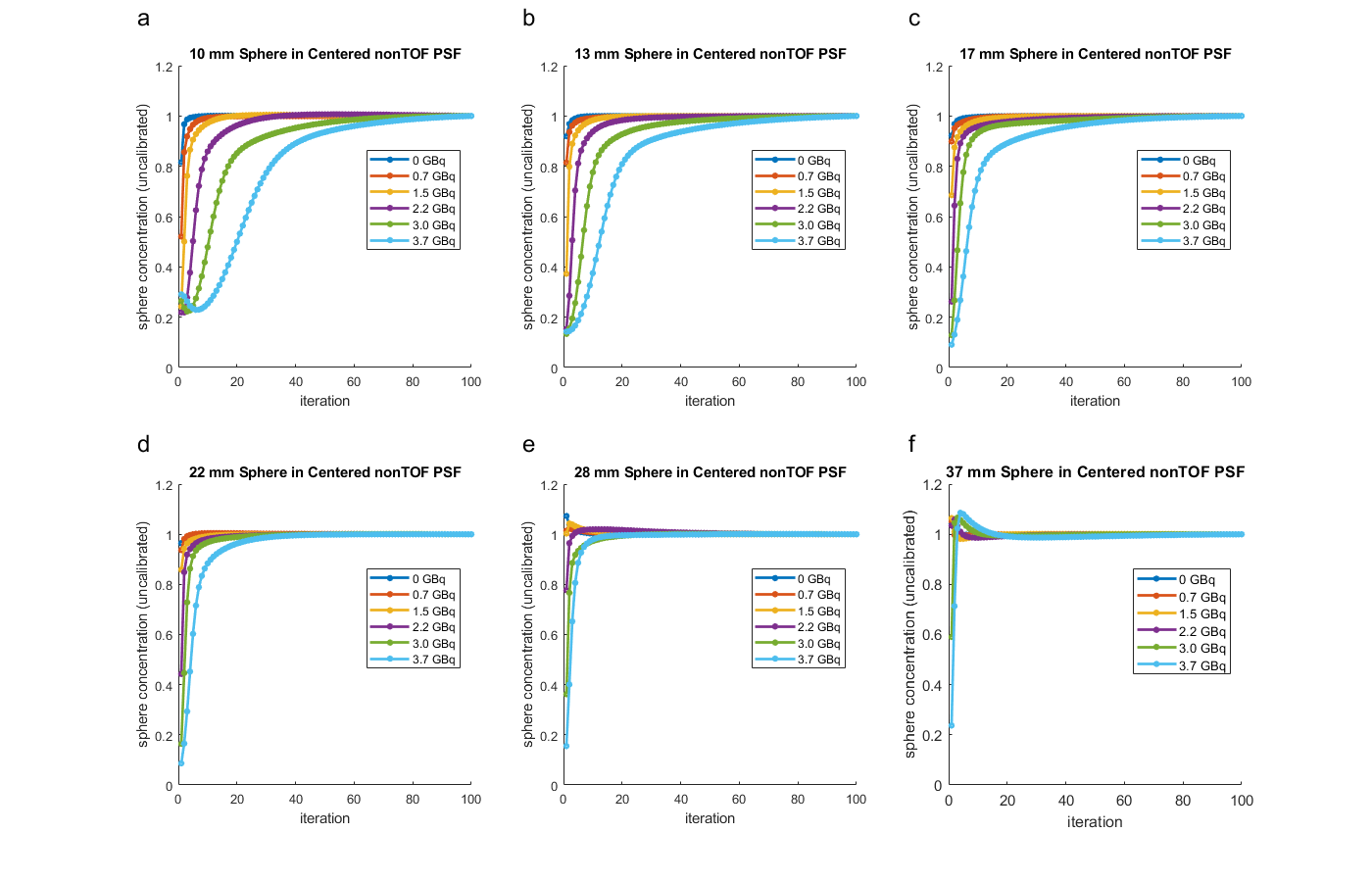

Supplement: Supplementary file 4 — Additional file 4: Figure S4. Same as Fig. S3 except for a centered phantom. [file 40658_2021_398_MOESM4_ESM.png]

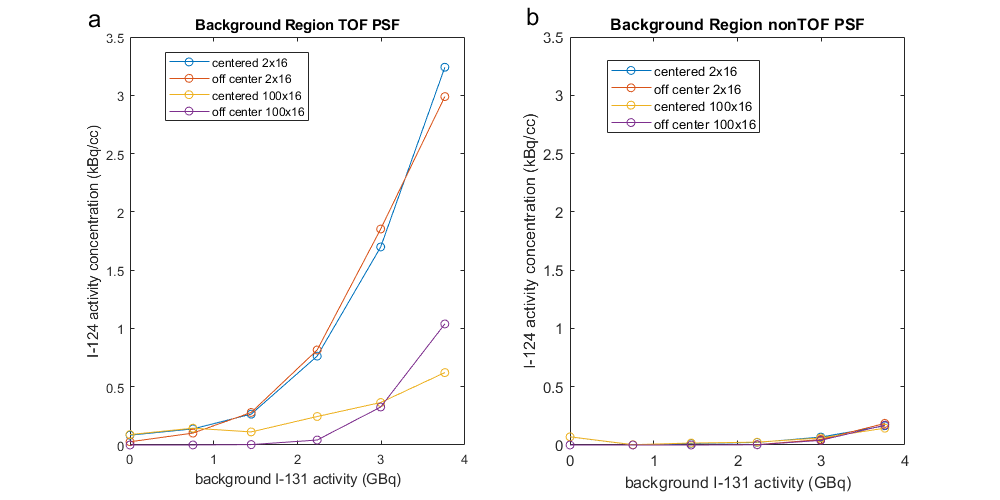

Supplement: Supplementary file 5 — Additional file 5: Figure S5. Background activity measured on the 124I PET image of the NEMA IEC phantom reconstructed with the OSEM algorithm with 2 and 100 iterations, as a function of increasing activity of 131I in the background and for the phantom centered and off centered within the field of view, using TOF information in (A) and without TOF information in (B). [file 40658_2021_398_MOESM5_ESM.png]

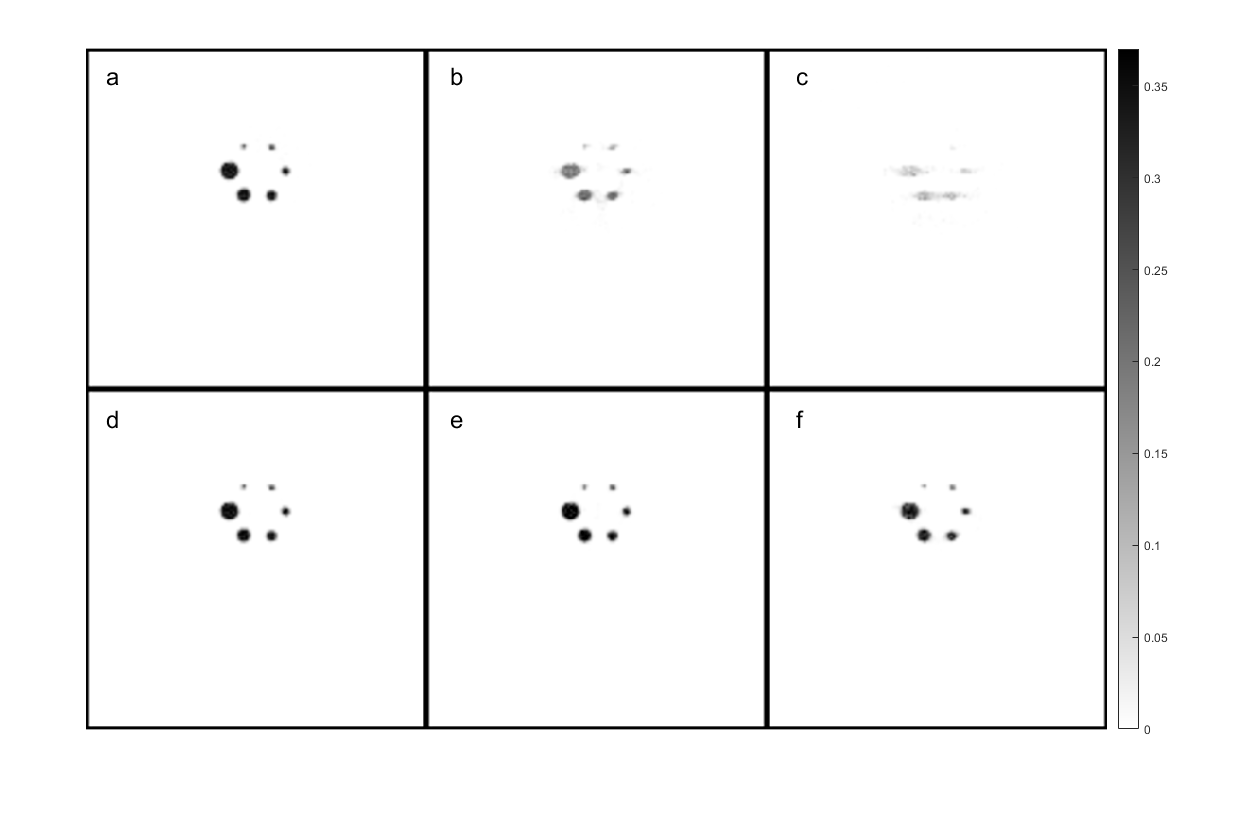

Supplement: Supplementary file 6 — Additional file 6: Figure S6. Transverse slice of the IEC phantom through the center of the hot spheres acquired with the phantom in the off-center position. The images on the top row (A, B, C) were reconstructed with OSEM using TOF information with 16 subsets and 100 iterations while the data was acquired with 2.2, 3.0 and 3.7 GBq of 131I in the background, respectively. In the second row (D, E, F) images were similarly reconstructed but without making use of TOF information. [file 40658_2021_398_MOESM6_ESM.png]

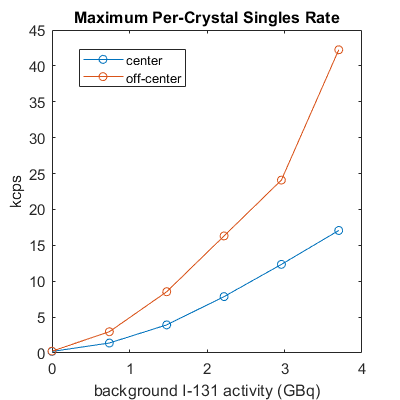

Supplement: Supplementary file 7 — Additional file 7: Figure S7. The maximum singles count rate of any crystal as a function of 131I in the phantom when centered in the FOV and when 11.2 cm off-center. [file 40658_2021_398_MOESM7_ESM.png]

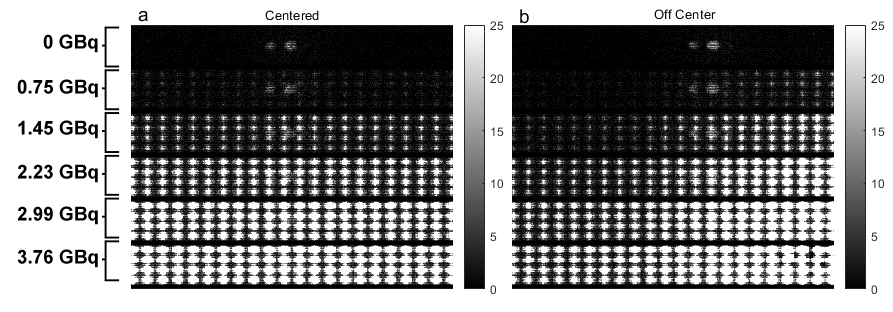

Supplement: Supplementary file 8 — Additional file 8: Figure S8. Projection image of the number of prompts for increasing 131I activity, for centered (A) and off-centered (B) acquisitions. [file 40658_2021_398_MOESM8_ESM.png]
